# Indication and electrical performance of conventional, resynchronization, and conduction system pacing in transthyretin amyloid cardiomyopathy

**DOI:** 10.1016/j.hroo.2026.03.026

**Published:** 2026-03-27

**Authors:** Stephan Dobner, Serlha Tawo, Fabian Noti, Fabian Wieser, Christoph Gräni, Christian Nitsche, Tobias Reichlin, Lukas Hunziker, Andreas Haeberlin

**Affiliations:** 1 Department of Cardiology, Inselspital, Bern University Hospital, University of Bern, Bern, Switzerland; 2 3rd Medical Department of Cardiology and Intensive Care Medicine, Klinik Ottakring (former Wilhelminenhospital), Vienna, Austria; 3 Medical Faculty, Sigmund Freud University, Vienna, Austria; 4 Ludwig Boltzmann Cluster for Cardiovascular Research, Vienna, Austria; 5 Division of Cardiology, Department of Internal Medicine II, Medical University of Vienna, Vienna, Austria

**Keywords:** Cardiac amyloidosis, Transthyretin, Pacemaker, Conduction pacing, Cardiac resynchronization therapy

## Abstract

**Background:**

Conduction system disorders and arrhythmias may require pacemaker (PM) or implantable cardioverter-defibrillator implantation in transthyretin amyloid cardiomyopathy (ATTR-CM). The optimal mode of pacing in ATTR-CM remains unknown.

**Objective:**

This study aimed to compare the electrical performance and clinical outcomes of conventional, resynchronization, and conduction system pacing (CSP) in ATTR-CM.

**Methods:**

In this observational study, the effects of cardiac pacing were investigated in 250 consecutive patients with ATTR-CM presenting at Inselspital Bern between June 2019 and February 2023.

**Results:**

During follow-up, 67 of 250 patients (26.8%) received a PM. Implantation of conventional single- (VVI) or dual-chamber (DDD) PMs was more common before ATTR-CM diagnosis (n = 17 of 25; 68%), and physiological pacing (cardiac resynchronization therapy [CRT]/CSP) was increasingly used thereafter (n = 24 of 42 [57.1%]). Sick sinus syndrome (n = 11 of 35 [31.4%] vs 6 of 32 [18.8%]) and higher-degree atrioventricular block (n = 20 of 35 [57.1%] vs 9 of 32 [28.1%]) were more common indications for VVI/DDD, with pursuit of a pace/ablate strategy (n = 12 of 32 [37.5%]) and heart failure (HF) (n = 5 of 32 [15.6%]) contributing significantly to implantation of CRT/CSP. QRS duration width was significantly lower with CSP (122 ms [interquartile range {IQR} 120–139]) than CRT (155 ms [IQR 141–160]) or VVI/DDD (160 ms [IQR 144–180]; *P* < .001). Although left ventricular/left bundle branch area pacing capture thresholds were significantly lower after CSP (0.75 V [IQR 0.5–1.2]) than CRT (1.3 V [IQR 1.0–1.6]; *P* = .049) at implantation, no significant differences for atrial or ventricular lead performance were observed during follow-up (*P* > .05). Patients referred for CRT/CSP had a higher incidence of HF (*P* = .014) and more common HF hospitalizations (*P* = .03); however, mortality was not significantly different (*P* = .28).

**Conclusion:**

Offering stable electrical performance and improved resynchronization, the effect of CSP on clinical outcomes warrants further exploration in ATTR-CM.

ClinicalTrials.gov identifier: NCT04776824

## Graphical abstract

**Figure:**
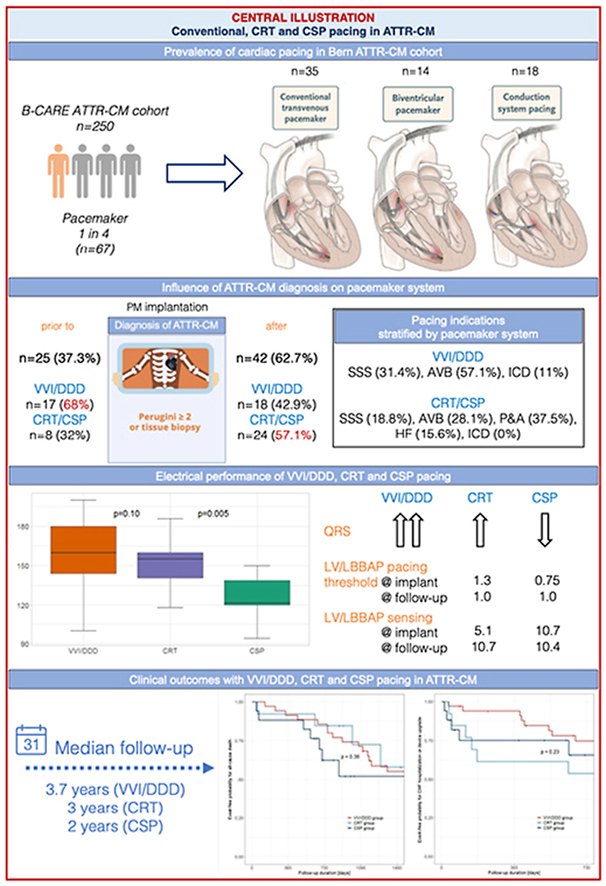

**Key Findings**

▪ The current study is the first to compare conventional, cardiac resynchronization therapy (CRT), and conduction system pacing (CSP) in transthyretin amyloid cardiomyopathy (ATTR-CM).
▪ After ATTR-CM diagnosis, indications for cardiac pacing change and with it the choice of pacemaker system, favoring CRT and CSP implantation to minimize the risk of heart failure progression previously described with high right ventricular pacing burden.
▪ Among all pacing modes, CSP resulted in improved electrical resynchronization with shorter paced QRS duration in patients with conduction disease, irrespective of intrinsic QRS morphology.
▪ After a median follow-up of almost 2 years, electrical lead performance after CSP implantation remained stable, without significant differences in sensing or pacing thresholds compared with conventional or CRT pacemakers.

## Introduction

Transthyretin amyloid cardiomyopathy (ATTR-CM) is a progressive protein misfolding and deposition disease that is increasingly recognized as an underlying cause of heart failure (HF) in elderly patients.^1^ Often preceding HF symptoms and thus providing an opportunity for early diagnosis and targeted therapy of ATTR-CM is an infiltration of the conduction system that may cause progressive conduction disorders requiring pacemaker (PM) implantation and also promote atrial fibrillation.^2^ Intricacies of cardiac pacing in ATTR-CM remain incompletely understood, and comparisons among the recently widely adopted left bundle branch area pacing (LBBAP), conventional pacing, and resynchronization pacing have not yet been performed. It has been suggested that high right ventricular (RV) pacing burden may be associated with worsening HF, which in contrast may be ameliorated by cardiac resynchronization therapy (CRT).^3^ Thus, a timely diagnosis of ATTR-CM before PM implantation may well influence the choice of a PM system. Moreover, atrial fibrillation is highly prevalent in ATTR-CM and an important driver of HF hospitalizations.^4^ With limited treatment options given that rate-controlling beta-blockers are poorly tolerated^5^ with advanced disease, a pace and ablate strategy is increasingly sought to alleviate HF symptoms and potentially improve clinical outcomes.

To help address current knowledge gaps, this study aimed to (1) assess how a diagnosis of ATTR-CM and the availability of cardiac resynchronization and conduction system pacing (CSP) influence PM indication and (2) to compare the early electrical performance of conventional, resynchronization, and CSP in patients with ATTR-CM.

## Methods

### Study population

Consecutive patients presenting with ATTR-CM at the Department of Cardiology, Bern University Hospital, Inselspital, Bern, Switzerland, between June 2019 and February 2023 were prospectively enrolled in the Bern Cardiac Amyloidosis Registry (NCT04776824) upon a written informed consent. Patients implanted with a cardiac PM before February 2023 were considered eligible for the study. Clinical data, including ATTR-CM–specific history, National Amyloidosis Centre disease stage, and electrocardiographic (ECG) and echocardiographic data, were collected from the time of PM implantation and the first and last available PM follow-up. PM indication and procedural details were recorded during the implantation procedure. Electrical performance was assessed at the time of PM implantation, and PM follow-up visits were conducted after 3 months and every 6–12 months thereafter. For analysis, performance measures at implantation and the last available PM follow-up visit were used. The study was approved by the local ethics committee (KEK: 2021-00135) and conducted according to the principles of the Declaration of Helsinki.

### ATTR-CM diagnosis

ATTR-CM diagnosis was made according to current international guidelines, as previously described.^4^ Noninvasively, if bone scintigraphy detected moderate or severe myocardial ^99m^Tc labeled 3,3-diphosphono-1,2-propanodicarboxylic acid tracer uptake (Perugini ≥2)^6^ after exclusion of light chain amyloidosis by a gammopathy panel and serum-free light chain assay.^7^ Alternatively, a biopsy-based diagnosis was made, with cardiac imaging (echocardiography or cardiac magnetic resonance imaging) required to confirm cardiac involvement in patients with extracardiac amyloid deposits.

### Implantation procedure and PM follow-up

Experienced electrophysiologists performed all device implantations. Patients were implanted with PMs/implantable cardioverter-defibrillators (ICDs) according to the current European Society of Cardiology guidelines on cardiac pacing. Whenever a CRT system was indicated, operators were free to implant an LBBAP lead instead of a conventional coronary sinus lead. Operators were also free to implant an LBBAP lead in lieu of a conventional RV lead.

If an LBBAP lead was implanted, successful conduction system activation was confirmed using established criteria for conduction system capture.^8^ Implanters were free to program base rates, tracking rates, ventricular sensing, and output according to clinical needs. Patients underwent device follow-up checks 3 months after the initial implant, then regular outpatient follow-up visits were scheduled every 6–12 months. During PM follow-up, lead parameters, including sensing (mV), impedance (Ω), and pacing thresholds (V/ms), were measured using the manufacturer’s interrogation device.

### ECG analysis

QRS duration of intrinsic and ventricular paced beats for all patient groups were measured manually using the ECGs before device implantation and during permanent ventricular pacing. All measurements were verified by a senior board-certified electrophysiologist.

### Statistical analysis

R version 4.1.2 for Windows (R Foundation, Vienna, Austria) was used for statistical analysis. Continuous variables are presented as mean ± standard deviation or median and interquartile range (IQR). Comparisons between groups were made using the Mann-Whitney U test (2 groups) or the Kruskal-Wallis test (3 groups). Categorical variables are expressed as numbers and percentages; comparisons were made with Fisher’s exact test. Electrode parameters after implant and during follow-up were compared using Wilcoxon’s signed-rank test. Comparisons in the occurrence of death, HF hospitalizations, or device upgrades were made using Kaplan-Meier estimates and compared using the log-rank test. A 2-sided *P* ≤ .05 was considered significant.

## Results

**Figure 1:**
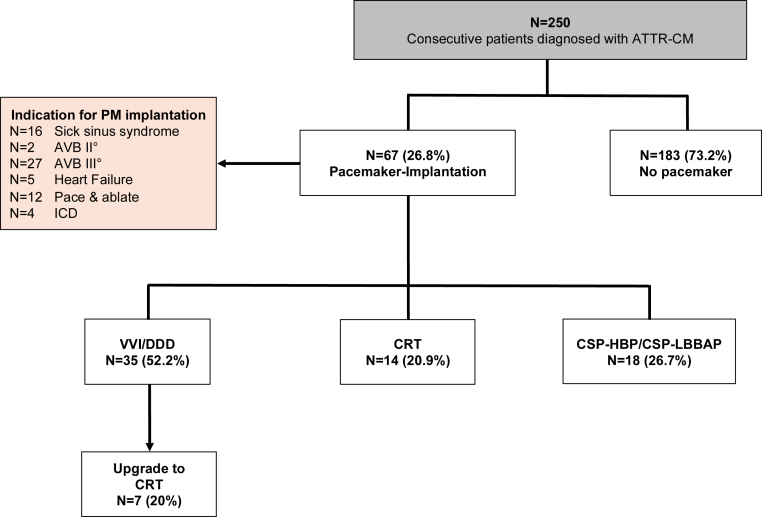
Study Consolidated Standards of Reporting Trials flowchart. ATTR-CM = transthyretin amyloid cardiomyopathy; AVB = atrioventricular block; CRT = cardiac resynchronization therapy; CSP = conduction system pacing; DDD = dual-chamber; HBP = His bundle pacing; ICD = implantable cardioverter-defibrillator; LBBAP = left bundle branch area pacing; TTR = transthyretin; VVI = single-chamber.

67 of 250 patients with ATTR-CM (26.8%) (Figure 1) presenting with ATTR-CM between June 2019 and February 2023 were implanted with a PM. PM implantation was performed before ATTR-CM diagnosis in 25 patients (37.3%) and thereafter in 42 patients (62.7%). A conventional single- (VVI) or dual-chamber (DDD) PM was implanted in 35 patients (52.2%), whereas 14 (20.9%) received a CRT PM and 18 (26.7%) a CSP device, respectively. Implantation of VVI or DDD PMs was common in undiagnosed ATTR-CM (n = 17 of 25; 68%), and physiological pacing systems (CRT/CSP) were implanted in most patients after diagnosis (n = 24 of 42; 57.1%). In yet to be diagnosed patients, PM implantation was performed a median of 19.9 months (IQR 6.0–54.4) before ATTR-CM diagnosis. CRT or CSP PM implantation occurred at a median of 22.1 (IQR −1.3 to 27.0) and 11.9 months (IQR 1.4–24.1), respectively, after ATTR-CM diagnosis (Table 1).

**Table 1:** Patient characteristics at the time of pacemaker implantation

Patient characteristics	VVI/DDD group, n = 35	CRT group, n = 14	CSP group, n = 18	*P* value
Age, y	81.6 (78.5–86.4)	79.4 (75.4–78.7)	81.1 (80.7–83.8)	.20
Male sex	34 (97)	13 (93)	16 (89)	.41
Coronary artery disease	17 (49)	4 (29)	6 (33)	.41
Valvular heart disease	13 (37)	4 (29)	3 (17)	.32
Diabetes mellitus	7 (20)	0 (0)	5 (28)	.11
Arterial hypertension	23 (66)	7 (50)	15 (83)	.13
Obstructive sleep apnea	4 (11)	3 (21)	1 (6)	.42
Atrial fibrillation	6 (17)	3 (21)	3 (17)	.82
ATTR-CM history				
Time since diagnosis (mo)	0.0 (−19.7 to 1.4)	22.1 (−1.3 to 27.0)	11.9 (1.4–24.1)	.005∗
Time on tafamidis (mo)	6.7 (5.3–23.3)	4.6 (3.0–8.0)	8.2 (4.0–11.1)	.23
Genetic testing performed	23 (66)	13 (93)	16 (89)	.028∗
ATTR disease stage at implant	2 (1–2)	3 (2–3)	2 (1–2)	.18
NAC stage I	7 (37)	3 (23)	5 (29)	
NAC stage II	9 (47)	3 (23)	8 (47)	
NAC stage III	3 (16)	7 (54)	4 (24)	
NYHA class at implant	2 (1–2)	3 (2–3)	2 (2–3)	.09
I	8 (28)	1 (7)	3 (17)	
II	14 (48)	5 (36)	10 (56)	
III	6 (21)	8 (57)	5 (28)	
IV	1 (3)	0 (0)	0 (0)	
Biomarkers				
eGFR (mL/min/1.73 m^2^)	57 (34–68)	37 (31–53)	43 (35–65)	.351
NT-proBNP (pg/mL)	2458 (1268–5186)	7806 (2997–10531)	2039 (1372–4807)	.06
Echocardiography				
LVEF (%)	58 (45–64)	30 (25–55)	55 (46–60)	.043∗
LV wall thickness (mm)	16 (15–18)	16 (16–19)	15 (14–17)	.49
LVEDD (mm)	45 (42–49)	48 (43–55)	44 (39–50)	.45
TAPSE (mm)	18 (15–20)	14 (10–16)	16 (11–20)	.12
TR degree	1 (1–2)	1 (1–1)	1 (1–2)	.77
MR degree	1 (1–2)	1 (1–1)	1 (1–1)	.45
sPAP estimated (mm Hg)	42 (36–50)	32 (26–47)	56 (45–70)	.18
Drug therapy				
ACEI	6 (17)	4 (29)	4 (22)	.57
ARB	14 (40)	5 (36)	6 (33)	.94
ARNI	1 (3)	4 (29)	1 (6)	.03∗
Beta-blocker	17 (49)	7 (50)	6 (33)	.55
MRA	7 (20)	8 (57)	9 (50)	.01∗
SGLT2-I	0 (0)	8 (57)	9 (50)	<.001∗
Torasemide equivalent (mg/d)	5 (0–10)	15 (1–24)	5 (5–10)	.18
Amiodarone	1 (3)	5 (36)	6 (33)	.002∗
OAC	19 (54)	12 (86)	14 (78)	.12
Tafamidis during study period	15 (43)	11 (79)	12 (67)	.07

Values are presented as median (interquartile range) or numbers (percentages). Comparisons between groups were made using Fisher’s exact test or the Kruskal-Wallis test as appropriate.

ACEI = angiotensin-converting enzyme inhibitor; ARB = angiotensin receptor blocker; ARNI = angiotensin receptor–neprilysin inhibitor; ATTR-CM = transthyretin amyloid cardiomyopathy; AVB = atrioventricular block; CRT = cardiac resynchronization therapy; CSP = conduction system pacing; DDD = dual-chamber; eGFR = estimated glomerular filtration rate; LBBB = left bundle branch block; LV = left ventricle; LVEDD = left ventricular end-diastolic diameter; LVEF = left ventricular ejection fraction; MR = mitral valve regurgitation; MRA = mineralocorticoid receptor antagonist; NAC = National Amyloidosis Centre; NT-proBNP = N-terminal pro-brain natriuretic peptide; NYHA = New York Heart Association; OAC = oral anticoagulation; PVI = pulmonary vein isolation; SGLT2-I = sodium-glucose transport protein 2 inhibitor; sPAP = systolic pulmonary artery pressure; TAPSE = tricuspid annular plane systolic excursion; TR = tricuspid valve regurgitation; VVI = single-chamber.

∗ Statistically significant.

### Baseline characteristics

The median age at implantation was 81.6 (78.5–86.4), 79.4 (75.4–78.7), and 81.1 years (80.7–83.8) for VVI/DDD, CRT, and CSP devices. Patients were predominantly male (n = 63 [94%]) (Table 1), and cardiovascular comorbidities were not significantly different between groups (Table 1). A significantly lower and severely depressed left ventricular ejection fraction (LVEF) was observed in CRT patients with 30% (IQR 25–55; *P* = .043), whereas LVEF was preserved at the time of implantation in VVI/DDD and CSP patients, respectively (58% [IQR 45–64]; 55% [IQR 46–60]). Numerically higher N-terminal pro-brain natriuretic peptide (NT-proBNP) levels were seen in the CRT group (7806 pg/mL [IQR 2997–10531]) than VVI/DDD and CSP patients (2458 pg/mL [IQR 1268–5186]; 2039 pg/mL [IQR 1372–4807]; *P* = .06). Furthermore, kidney function measured by estimated glomerular filtration rate (eGFR) was lower in CRT patients, albeit nonsignificantly with 37 mL/min/1.73 m^2^ (IQR 31–51), than those implanted with a VVI/DDD or CSP device with 57 mL/min/1.73 m^2^ (IQR 34–68) and 43 mL/min/1.73 m^2^ (IQR 35–65), respectively (*P* = .35).

When comparing baseline medication at implantation, significantly higher rates of mineralocorticoid receptor antagonists (*P* = .01) (Table 1) and sodium-glucose transport protein 2 inhibitors (*P* < .001) (Table 1) were recorded in patients receiving a CRT or CSP PM than those implanted with a VVI/DDD PM. Prescription rates of renin-angiotensin-aldosterone system inhibitors were highest for CRT patients (n = 13 of 14 [92.6%]; VVI/DDD n = 21 of 35 [60%]; CSP n = 11 of 18 [61%]), with a significantly higher proportion of CRT candidates on angiotensin receptor–neprilysin inhibitors (*P* = .03) (Table 1). During the study period, transthyretin (TTR)-stabilizing therapy was prescribed in 43% of patients receiving a VVI/DDD PM compared with 79% and 67% implanted with a CRT and CSP device, respectively (*P* = .07).

### PM indication and procedural and electrical outcomes at PM implantation

Sick sinus syndrome (SSS) (11 of 35 [31.4%] vs 6 of 32 [18.8%]) and higher-degree atrioventricular (AV) block (20 of 35 [57.1%] vs 9 of 32 [28.1%]) were more common indications for VVI/DDD PM implantation than CRT/CSP PMs. Pursuit of a pace/ablate strategy (12 of 32; 37.5%; *P* < .001) and clinically manifest HF (5 of 32; 15.6%; *P* = .017) triggered implantation of CRT/CSP PMs (Table 2). Primary prophylactic ICD implantation was performed in 4 patients (11%) in the VVI/DDD group, yet this was not an indication in any of the CRT/CSP patients (*P* = .17).

**Table 2:** PM indication and procedural and electrical outcomes after PM implantation in patients with ATTR-CM

	VVI/DDD group, n = 35	CRT group, n = 14	CSP group, n = 18	*P* value
Implant indication				
Sick sinus syndrome	11 (31)	2 (14)	4 (22)	.46
AVB I	11 (31)	5 (36)	13 (72)	.09
AVB II	2 (6)	0 (0)	0 (0)	.72
Paroxysmal AVB III	10 (29)	1 (7)	4 (22)	.29
Permanent AVB III	8 (23)	2 (14)	2 (11)	.64
Paroxysmal/permanent AVB III	18 (52)	3 (21)	6 (33)	.15
Pace and ablate strategy	0 (0)	6 (43)	6 (33)	<.001∗
Heart failure	0 (0)	3 (21)	2 (11)	.017∗
ICD indication	4 (11)	0 (0)	0 (0)	.17
Duration of implantation (min)	60 (52–92)	140 (115–208)	125 (85–180)	<.001∗
Fluoroscopy duration (min)	7 (5–10)	26 (20–48)	17 (9–25)	<.001∗
Radiation dose (cGy × cm^2^)	257 (67–1129)	1694 (968–3738)	507 (304–1709)	.008∗
Intrinsic QRS duration (ms)	138 (123–150)	144 (130–160)	139 (112–158)	.80
Paced QRS duration (ms)	160 (144–180)	155 (141–160)	122 (120–139)	<.001∗
QRS lengthening by pacing (ms)	19 (−4 to 50)	6 (−24 to 15)	−1.5 (−28 to 19.8)	.025∗
Base rate (bpm)	60 (60–60)	60 (53–68)	60 (60–68)	.26
Maximum tracking rate (bpm)	130 (130–130)	130 (130–133)	130 (130–130)	.20
Electrode parameters				
RA sensing (mV)	2.1 (1.4–2.8)	1.8 (1.0–4.5)	1.5 (1.2–2.9)	.81
RA threshold (V/0.4 ms)	0.73 (0.5–1.13)	0.6 (0.5–0.8)	0.5 (0.45–0.7)	.36
RA impedance (Ω)	507 (440–549)	536 (440–611)	542 (492–597)	.74
RV sensing (mV)	9.6 (6.2–13.8)	8.9 (5.8–12.0)	7.2 (5.0–9.6)	.45
RV impedance (Ω)	676 (554–757)	515 (432–788)	526 (507–589)	.17
RV threshold (V/0.4 ms)	0.5 (0.39–0.65)	0.5 (0.5–0.6)	0.5 (0.5–0.7)	.66
LV/LBBAP sensing (mV)	-	5.1 (4.8–7.9)	10.7 (7.9–14.6)	.22
LV/LBBAP threshold (V/0.4 ms)	-	1.3 (1.0–1.6)	0.75 (0.5–1.2)	.049∗
LV/LBBAP impedance (Ω)	-	494 (435–835)	565 (420–627)	.53

Values are presented as median (interquartile range) or numbers (percentages). Comparisons between groups were made using the Kruskal-Wallis test or the Mann-Whitney U test as appropriate.

ATTR-CM = transthyretin amyloid cardiomyopathy; AVB = atrioventricular block; bpm = beats/min; CRT = cardiac resynchronization therapy; CSP = conduction system pacing; DDD = dual-chamber; ICD = implantable cardioverter-defibrillator; LBBAP = left bundle branch area pacing; LV = left ventricle; PM = pacemaker; RA = right atrium; RV = right ventricle; VVI = single-chamber.

∗ Statistically significant.

Implant duration was shortest in the VVI/DDD PM group with 60 minutes (IQR 52–92) compared with 125 (IQR 85–180) and 140 minutes (IQR 115–208) in the CSP and CRT groups, respectively (*P* < .001) (Table 2). Accordingly, fluoroscopy time was shortest (7 minutes [IQR 5–10]) and radiation dose lowest (257 cGy × cm^2^ [IQR 67–1129]) if VVI/DDD PMs were implanted. Compared with CRT implantation, fluoroscopy time in the CSP group was significantly shorter (17 minutes [IQR 9–25] vs 26 [IQR 20–48]; *P* = .03) and radiation exposure significantly lower (507 cGy × cm^2^ [IQR 304–1709] vs 1694 [IQR 968–3738]; *P* = .02).

**Figure 2:**
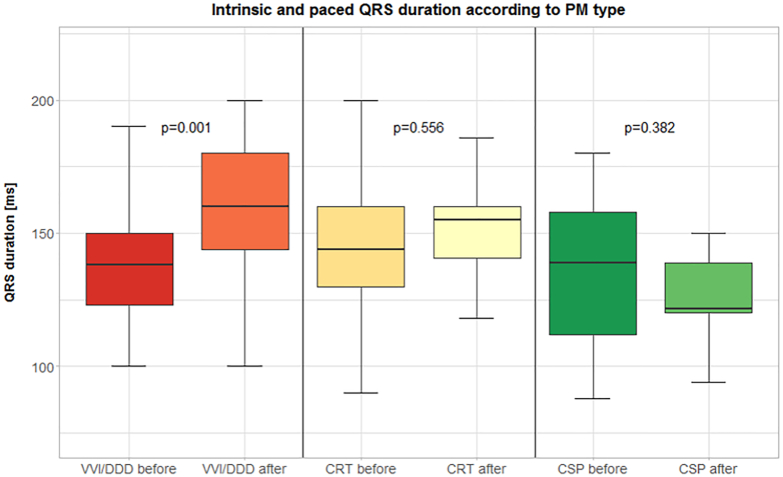
Intrinsic vs paced QRS duration according to the PM system in patients with cardiac amyloidosis. CRT = cardiac resynchronization therapy; CSP = conduction system pacing; DDD = dual-chamber; PM = pacemaker; VVI = single-chamber.

**Figure 3:**
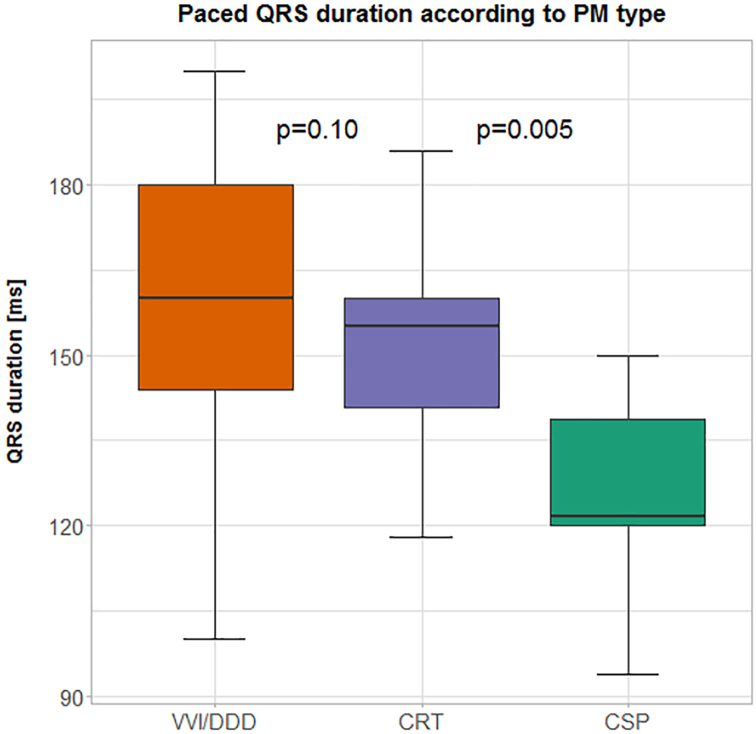
Paced QRS duration according to the PM system in patients with cardiac amyloidosis. CRT = cardiac resynchronization therapy; CSP = conduction system pacing; DDD = dual-chamber; PM =pacemaker; VVI = single-chamber.

Although intrinsic QRS duration was comparable between groups, it increased after VVI/DDD PM (+19 ms [IQR −4 to 50]) and CRT implantation (+6 ms [IQR −24 to 15]), while decreasing after CSP PM placement (−1.5 ms [IQR −28 to −19.8]; *P* = .025) (Table 2; Figure 2). Paced QRS duration was significantly shorter after CSP implantation with 122 ms (IQR 120–139) compared to VVI/DDD (160 ms [IQR 144–180]) and CRT pacing (155 ms [IQR 141–160]; *P* < .001) (Table 2; Figure 3).

Left ventricular (LV)/LBBAP capture thresholds (0.4 ms pulse width) were significantly lower after CSP (0.75 V [IQR 0.5–1.2]) than CRT (1.3 V IQR [1.0–1.6]; *P* = .049). At implant, right atrium (RA) and RV lead performance were comparable across all pacing systems (*P* > .05).

### Effect of pacing mode on QRS duration according to preimplantation bundle branch block subtype

Left bundle branch block (LBBB) was the most common conduction disorder present in patients implanted with a CRT device (n = 5 [45%]; *P* = .006) (Table 3). The QRS width was >130 ms in all 5 CRT candidates with LBBB, with 80% (n = 4) presenting with a QRS of >150 ms before implantation. Right bundle branch block was significantly more common in patients implanted with a VVI/DDD PM (n = 14 [52%]; *P* = .011) (Table 3).

**Table 3:** Intrinsic conduction system disorders in patients implanted with VVI/DDD, CRT, and CSP pacemakers

Native QRS morphology	VVI/DDD group, n = 35	CRT group, n = 14	CSP group, n = 18	*P* value
Narrow complex	4 (15)	2 (18)	5 (28)	.006∗
LBBB	6 (22)	5 (45)	6 (33)	.006∗
LBBB >150 ms	1 (4)	4 (36)	3 (17)	
LBBB 130–150 ms	4 (15)	1 (9)	2 (11)	
LBBB <130 ms	1 (4)	0 (0)	1 (6)	
RBBB	14 (52)	3 (27)	6 (33)	.011∗
RBBB >150 ms	4 (15)	0 (0)	3 (17)	
RBBB 130–150 ms	6 (22)	3 (27)	3 (17)	
RBBB <130 ms	2 (7)	0 (0)	0 (0)	
IVCD	3 (11)	1 (9)	1 (6)	<.001∗

Values are presented as numbers (percentages). Comparisons between groups were made using Fisher’s exact test.

CRT = cardiac resynchronization therapy; CSP = conduction system pacing; DDD = dual-chamber; IVCD = intraventricular conduction delay; LBBB = left bundle branch block; RBBB = right bundle branch block; VVI = single-chamber.

∗ Statistically significant.

Irrespective of the intrinsic QRS morphology and duration, implantation of a VVI/DDD PM increased paced QRS duration (Table 4). CRT implantation numerically reduced paced compared with intrinsic QRS duration in patients presenting with preimplantation LBBB or intraventricular conduction delay, whereas a prolongation was seen in patients with narrow intrinsic QRS or right bundle branch block morphology (Table 4). Regardless of conduction system disorder and QRS morphology at implantation, CSP implantation led to a numerical reduction in paced QRS duration (Table 4).

**Table 4:** Intrinsic and paced QRS duration according to bundle branch block subtype and CIED

Native QRS morphology	VVI/DDD group, n = 35			CRT group, n = 14			CSP group, n = 18		*P* value
	Intrinsic QRS duration (ms)	Paced QRS duration (ms)	*P* value	Intrinsic QRS duration (ms)	Paced QRS duration (ms)	*P* value	Intrinsic QRS duration (ms)	Paced QRS duration (ms)	
Narrow complex	104 (93–107)	150 (130–159)	.50	103 (96–109)	137 (134–141)	.50	94 (89–95)	120 (120–120)	.06
LBBB	150 (135–150)	178 (149–199)	.13	200 (160–200)	160 (160–160)	.13	155 (139–170)	135 (123–147)	.53
RBBB	150 (134–164)	160 (154–174)	.17	140 (138–143)	160 (152–160)	.25	151 (143–158)	130 (121–139)	.06
IVCD	130 (130–175)	180 (168–182)	.75	144 (144–144)	120 (120–120)	1	124 (124–124)	120 (120–120)	1

Values are presented as median (interquartile range). Comparisons between groups were made using Wilcoxon’s signed-rank test.

CIED = cardiac implantable electronic device; CRT = cardiac resynchronization therapy; CSP = conduction system pacing; DDD = dual-chamber; IVCD = intraventricular conduction delay; LBBB = left bundle branch block; RBBB = right bundle branch block; VVI = single-chamber.

### Electrical performance of VVI/DDD, CRT, and CSP PMs during follow-up

The median time for the last available PM follow-up was 1341 (IQR 881–2186), 1084 (IQR 911–1501), and 714 days (IQR 592–981) after VVI/DDD, CRT, and CSP PM implantation, respectively (Table 5). Pacing burden was high, at 94% (IQR 69–99), 99% (IQR 99–99), and 99% (IQR 95–99) for VVI/DDD, CRT, and CSP PMs, respectively.

**Table 5:** Electrical lead performance and pacing burden during follow-up according to pacing mode in patients with ATTR-CM

	VVI/DDD group, n = 35			CRT group, n = 14			CSP group, n = 18		
Median follow-up duration, d	1341 (881–2186)			1084 (911–1501)			714 (592–981)		.018∗
Ventricular pacing during FU, %	94 (69–99)			99 (99–99)			99 (95–99)		.028∗
Electrode parameters	At implant	During FU	*P* value	At implant	During FU	*P* value	At implant	During FU	*P* value
RA sensing (mV)	2.1 (1.4–2.8)	1.7 (1.0–3.3)	.097	1.8 (1.0–4.5)	1.7 (0.8–7.5)	1.000	1.5 (1.2–2.9)	2.3 (1.1–3.7)	.383
RA impedance (Ω)	507 (440–549)	460 (420–526)	.025∗	536 (440–611)	649 (480–684)	.438	542 (492–597)	527 (482–646)	.182
RA threshold (V/0.4 ms)	0.73 (0.5–1.1)	0.75 (0.7–1.0)	.297	0.6 (0.5–0.8)	0.75 (0.7–0.8)	.750	0.5 (0.5–0.7)	0.9 (0.8–1.5)	.063
RV sensing (mV)	9.6 (6.2–13.8)	6.5 (4.7–8.7)	.081	8.9 (5.8–12.0)	5.7 (2.5–7.2)	.578	7.2 (5.0–9.6)	10.3 (7.8–16.5)	.375
RV impedance (Ω)	676 (554–757)	460 (410–519)	<.001∗	515 (432–788)	531 (410–638)	.137	526 (507–589)	526 (468–550)	.063
RV threshold (V/0.4 ms)	0.5 (0.4–0.7)	0.8 (0.7–1.0)	<.001∗	0.5 (0.5–0.6)	0.7 (0.7–0.8)	.020∗	0.5 (0.5–0.7)	1.1 (0.8–1.4)	.016∗
LV/LBBAP sensing (mV)	-	-		5.1 (4.8–7.9)	10.65 (6.6–14.7)	1.000	10.7 (7.9–14.6)	10.4 (9.0–13.8)	.250
LV/LBBAP impedance (Ω)	-	-		494 (435–835)	674 (569–855)	.359	565 (420–627)	437 (344–546)	.570
LV/LBBAP threshold (V/0.4 ms)	-	-		1.3 (1.0–1.6)	1.0 (0.8–1.3)	.25	0.8 (0.5–1.2)	1.0 (0.6–1.3)	.500

Values are presented as median (interquartile range). Comparisons between groups were made using Wilcoxon’s signed-rank test.

ATTR-CM = transthyretin amyloid cardiomyopathy; CRT = cardiac resynchronization therapy; CSP = conduction system pacing; DDD = dual-chamber; FU = follow-up; LBBAP = left bundle branch area pacing; LV = left ventricle; RA = right atrium; RV = right ventricle; VVI = single-chamber.

∗ Statistically significant.

At follow-up, no significant lead performance differences were seen between PM systems (Supplemental Table 1). Atrial, RV, and LV/LBBAP sensing did not significantly change compared with baseline during follow-up, irrespective of the PM system implanted (*P* > .05 for all) (Table 5). When comparing lead performance with the time of implantation, significantly lower RA and RV lead impedance were observed at the last follow-up for VVI/DDD PMs (RA 460 Ω [420–526]; *P* = .025; RV 460 Ω [410–519]; *P* < .001) (Table 5). Significant, but not clinically relevant, increases in RV pacing thresholds were seen in all PM systems (*P* < .05 for all). Although LBBAP capture threshold was lower at baseline than CRT LV thresholds (0.75 V/0.4 ms [IQR 0.5–1.2] vs 1.3 V/0.4 ms [IQR 1.0–1.6]; *P* = .049), performance at follow-up was not significantly different (1.0 V/0.4 ms [IQR 0.6–1.3] vs 1.0 V/0.4 ms [IQR 0.8–1.3]; *P* > .05).

### Effect of VVI/DDD pacing, CRT pacing, and CSP on clinical outcomes

**Figure 4:**
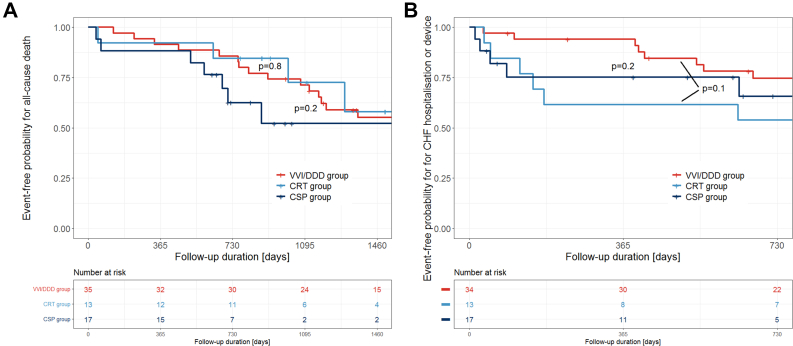
Kaplan-Meier survival estimates for all-cause mortality (**A**) and a combined endpoint of heart failure hospitalization or device upgrade (**B**). Patients at risk shown on the x-axis. CHF = congestive heart failure; CRT = cardiac resynchronization therapy; CSP = conduction system pacing; DDD = dual-chamber; VVI = single-chamber.

The median follow-up time for clinical outcomes from device implantation was 1341 days (IQR 881–2186), 1084 (IQR 911–1501), and 714 days (IQR 592–981) for patients implanted with VVI/DDD, CRT, and CSP PMs, respectively (Table 6). Overall, 32 patients died during follow-up (Table 6). Annualized mortality was 15.2%, 12.0%, and 19.9% for VVI/DDD, CRT, and CSP patients, respectively (Table 6). Time to first event analysis did not show a significant effect of the pacing modality on mortality (*P* = .38 [across all 3 groups]; *P* = .2 [VVI/DDD vs CSP]) (Figure 4A).

**Table 6:** Clinical outcomes, functional status, cardiorenal biomarkers, and echocardiographic follow-up according to pacing mode in patients with ATTR-CM

	VVI/DDD group, n = 35	CRT group, n = 14	CSP group, n = 18	*P* value
Median follow-up duration for CV outcomes, d	1341 (881–2186)	1084 (911–1501)	714 (592–981)	.018∗
Any HFH	13 (37)	11 (79)	12 (67)	.014∗
Number of HFH, n	31	29	27	.030∗
Annualized HFH rate (%)	24.1	69.7	76.7	
Death	20 (57)	5 (36)	7 (39)	.28
Annualized death rate (%)	15.2	12.0	19.9	
Median follow-up duration for clinical status, biomarkers, TTE, d	1002 (719–1532)	477 (209–708)	251 (94–345)	<.001∗
Clinical characteristics at FU				
NYHA class	2 (1–2)	2 (1–2)	2 (2–3)	.54
Diuretic, torsemide equivalent (mg/d)	7.5 (3–19)	10 (9–22)	6 (5–10)	.65
Biomarkers				
eGFR (mL/min per 1.73 m^2^)	39 (33–61)	43 (35–60)	38 (29–49)	.65
NT-proBNP (pg/mL)	2822 (1406–5588)	4669 (2682–6526)	5915 (1338–6347)	.64
TTE parameters during FU				
LVEF (%)	49 (40–54)	48 (30–55)	55 (36–60)	.64
LVEDD (mm)	48 (43–51)	44 (36–52)	43 (39–47)	.23
TAPSE (mm)	15 (12–17)	16 (11–17)	18 (12–21)	.50
TR degree	1 (1–1)	1 (1–1)	1 (1–2)	.69
MR degree	1 (1–1)	1 (1–1)	1 (1–2)	.11
sPAP estimated (mm Hg)	42 (35–51)	31 (29–32)	33 (32–34)	.014∗

Values are presented as median (interquartile range) or numbers (percentages). Comparisons between groups were made using the Kruskal-Wallis test or the Mann-Whitney U test as appropriate.

ATTR-CM = transthyretin amyloid cardiomyopathy; CRT = cardiac resynchronization therapy; CSP = conduction system pacing; CV = cardiovascular; DDD = dual-chamber; eGFR = estimated glomerular filtration rate; FU = follow-up; HFH = heart failure hospitalization; LVEDD = left ventricular end-diastolic diameter; LVEF = left ventricular ejection fraction; MR = mitral valve regurgitation; NT-proBNP = N-terminal pro-brain natriuretic peptide; NYHA = New York Heart Association; sPAP = systolic pulmonary artery pressure; TAPSE = tricuspid annular plane systolic excursion; TR = tricuspid valve regurgitation; TTE = transthoracic echocardiogram; VVI = single-chamber.

∗ Statistically significant.

HF hospitalizations were recorded for 37% (n = 13), 79% (n = 11), and 67% of patients (n = 12) after VVI/DDD, CRT, and CSP implantation (*P* = .014) (Table 6), respectively, translating into annualized hospitalization rates of 24.1% (VVI/DDD), 69.7% (CRT), and 76.7% (CSP) (Table 6). Time to first event analysis for HF events or the need for device upgrade did not show a significant effect of the pacing modality during follow-up (*P* = .23 [across all 3 groups]; *P* = .2 [VVI/DDD vs CRT]; *P* = .1 [VVI/DDD vs CSP]) (Figure 4B).

### Effect of VVI/DDD pacing, CRT pacing, and CSP on cardiorenal biomarkers and echocardiographic parameters

The median follow-up time from device implantation for clinical status, cardiorenal biomarkers, and echocardiographic parameters was 1002 (IQR 719–1532), 477 (IQR 209–708), and 251 days (IQR 94–345) for patients implanted with VVI/DDD, CRT, and CSP PMs, respectively (*P* < .001) (Table 6). Differences in New York Heart Association (NYHA) class (*P* = .54) or daily diuretic dosage were not seen at follow-up between groups (*P* = .65) (Table 6). When comparing within-group differences from the time of PM implantation to the last follow-up, a minor increase in diuretic dose was observed in VVI/DDD patients (from 5 [0–10] to 7.55 [5–10] mg torsemide equivalent; *P* = .02) (Table 7), whereas dosages did not significantly differ in the other 2 groups. NYHA functional class improved during follow-up after CRT implantation (*P* = .027) (Table 7); no significant effect was observed in patients receiving VVI/DDD or CSP PMs.

**Table 7:** Clinical, laboratory, and echocardiographic parameters at baseline and follow-up according to pacing mode in patients with ATTR-CM

	VVI/DDD group, n = 35			CRT group, n = 14			CSP group, n = 18		
	At implantation	At the last FU	*P* value	At implantation	At the last FU	*P* value	At implantation	At the last FU	*P* value
Clinical characteristics at FU		n = 17			n = 12			n = 13	
ATTR disease stage			.48			1			.71
NAC stage I	7 (37)	7 (41)		3 (23)	3 (25)		5 (29)	2 (15)	
NAC stage II	9 (47)	5 (29)		3 (23)	3 (25)		8 (47)	7 (54)	
NAC stage III	3 (16)	5 (29)		7 (54)	6 (50)		4 (24)	4 (31)	
NYHA class			.97			.027∗			1
I	8 (28)	10 (32)		1 (7)	4 (33)		3 (17)	3 (21)	
II	14 (48)	14 (45)		5 (36)	7 (58)		10 (56)	7 (50)	
III	6 (21)	6 (19)		8 (57)	1 (8)		5 (28)	4 (29)	
IV	1 (3)	1 (3)		0 (0)	0 (0)		0 (0)	0 (0)	
Diuretic, torsemide equivalent (mg/d)	5 (0–10)	7.55 (5–10)	.02∗	15 (1–24)	10 (9–23)	.69	5 (5–10)	6 (5–10)	.31
Biomarkers	n = 23	n = 17		n = 13	n = 12		n = 18	n = 13	
eGFR (mL/min/1.73 m^2^)	57 (34–68)	39 (33–61)	.02∗	37 (31–53)	43 (35–60)	.65	43 (35–65)	38 (29–49)	.04∗
NT-proBNP (pg/mL)	2458 (1268–5186)	2822 (1406–5588)	.25	7806 (2997–10531)	4669 (2682–6526)	.70	2039 (1372–4807)	5915 (1338–6347)	.08
TTE parameters during FU	n = 31	n = 30		n = 14	n = 12		n = 18	n = 14	
LVEF (%)	58 (45–64)	49 (40–54)	.10	30 (25–55)	48 (30–55)	.87	55 (46–60)	55 (36–60)	.45
LVEDD (mm)	45 (42–49)	48 (43–51)	.35	48 (43–55)	44 (36–52)	.92	44 (39–50)	43 (39–47)	.44
TAPSE (mm)	18 (15–20)	15 (12–17)	.14	14 (10–16)	16 (11–17)	.25	16 (11–20)	18 (12–21)	.77
TR degree			.81			1			.67
I	15 (48)	20 (67)		8 (57)	8 (67)		8 (44)	7 (50)	
II	3 (10)	3 (10)		1 (7)	2 (17)		3 (17)	5 (36)	
III	4 (13)	3 (10)		1 (7)	1 (8)		0 (0)	0 0 (0)	
MR degree			<.001∗			.01∗			.16
I	17 (54)	0 (0)		9 (64)	2 (17)		11 (61)	7 (50)	
II	6 (19)	24 (80)		3 (21)	9 (75)		1 (6)	4 (29)	
III	0 (0)	3 (10)		0 (0)	1 (8)		0 (0)	1 (7)	
sPAP estimated (mm Hg)	42 (36–50)	42 (35–51)	.63	32 (26–47)	31 (29–32)	.50	56 (45–70)	33 (32–34)	1

Values are presented as median (interquartile range) or numbers (percentages). Comparisons were made using Fisher’s exact test or Wilcoxon’s signed-rank test as appropriate.

ATTR-CM = transthyretin amyloid cardiomyopathy; CRT = cardiac resynchronization therapy; CSP = conduction system pacing; DDD = dual-chamber; eGFR = estimated glomerular filtration rate; FU = follow-up; LVEDD = left ventricular end-diastolic diameter; LVEF = left ventricular ejection fraction; MR = mitral valve regurgitation; NAC = National Amyloidosis Centre; NT-proBNP = N-terminal pro-brain natriuretic peptide; NYHA = New York Heart Association; sPAP = systolic pulmonary artery pressure; TAPSE = tricuspid annular plane systolic excursion; TR = tricuspid valve regurgitation; TTE = transthoracic echocardiogram; VVI = single-chamber.

∗ Statistically significant.

At the last follow-up, eGFR for patients implanted with a VVI/DDD, CRT, or CSP PM was 39 mL/min/1.73 m^2^ (IQR 33–61), 43 mL/min/1.73 m^2^ (IQR 35–60), and 38 mL/min/1.73 m^2^ (IQR 29–49), respectively, and did not significantly differ (*P* = .65) (Table 6). When comparing eGFR from baseline with the last follow-up, a significant decrease in eGFR was seen in patients implanted with a VVI/DDD or CSP PM (*P* = .02 and *P* = .04) (Table 7), whereas no significant differences were observed after CRT implantation (*P* = .65) (Table 7). NT-proBNP levels did not significantly differ between groups at the last follow-up (*P* = .64) (Table 6). NT-proBNP numerically increased during follow-up after VVI/DDD and CSP implantation to 2822 (1406–5588) and 5915 pg/mL (1338–6347), respectively, whereas a numerical reduction to 4669 pg/mL (2682–6526) was seen after CRT implantation (*P* > .05 within all groups) (Tables 6 and 7).

LVEF at baseline was significantly lower in CRT candidates (30% [IQR 25–55]; *P* = .043) (Table 1), yet, after a numerical increase in LVEF to 48% (30–55) (*P* = .87) (Table 7), no significant differences were seen between groups at follow-up (*P* = .64) (Table 6). An increase in moderate mitral regurgitation was observed in all groups at follow-up (Table 7), reaching statistical significance in the VVI/DDD (*P* < .001) and CRT groups (*P* = .01) (Table 7).

## Discussion

In this study, we present the first comparison of conventional pacing, CRT pacing, and CSP in patients with ATTR-CM. The main findings are as follows:

1.) CSP was safely performed in this patient cohort, which is often challenging to implant.
2.) CSP resulted in shorter paced QRS complexes than conventional and CRT systems with consistently low LV pacing thresholds during a median follow-up of ∼2 years. Compared with CRT, implantation time and periprocedural radiation exposure were lower in the CSP group.
3.) After ATTR-CM diagnosis, HF and implantation of CRT/CSP PMs were more common. Despite favorable early electric results, the effect of CSP and alternative pacing strategies on clinical outcomes remains uncertain and needs further investigation.

Morbidity and mortality in ATTR-CM remain high, irrespective of TTR-targeting therapies. Progressive conduction system disease and arrhythmias present a significant clinical challenge, often requiring PM implantation, yet uncertainty remains high regarding the optimal pacing strategy. Based on current evidence, ATTR-CM diagnosis and the presence of HF both influenced the indication for cardiac pacing and the choice of the PM system implanted in our cohort. Before ATTR-CM diagnosis, 2 in 3 patients requiring cardiac pacing—mostly owing to higher-degree AV block (58%) and SSS (31%)—were implanted with conventional VVI/DDD PMs. After diagnosis, with a more common history of HF, CRT and CSP were favored and implanted in 57.1% of patients to minimize the risk of HF progression previously described with high RV pacing burden.^3^

Implantation time was shortest for VVI/DDD PMs, although not significantly different between CRT and CSP (*P* > .05). When comparing the 2 physiological pacing techniques, fluoroscopy duration and radiation exposure were significantly lower for CSP than CRT PM implantation. VVI/DDD PMs significantly and CRT numerically increased, whereas CSP PMs reduced QRS duration, resulting in significantly shorter QRS width after LBBAP implantation. Electrical lead performance was generally good at implantation and throughout follow-up. Low sensing during device implantation may present a challenge,^9^ yet LBBAP lead sensing was numerically higher than LV sensing at implant and remained stable during follow-up. At implantation, LBBAP threshold was significantly lower in LBBAP than LV pacing threshold in CRT patients, with thresholds remaining low. Although no significant differences for pacing thresholds were seen during follow-up, improved early thresholds may yet translate into improved battery longevity.

Increasing clinical evidence suggests that LBBAP may emerge as the preferred pacing methodology for most patients in need of cardiac pacing. Improvements in LVEF, beneficial cardiac remodeling with a reduction in LV diameters, mitral regurgitation, and natriuretic peptide levels have been described with LBBAP,^10^ and even in CRT nonresponders, upgrade to LBBAP may be associated with improved functional capacity, increased LVEF, and a reduction after HF hospitalizations.^11^ However, with limited available evidence, particularly for clinical outcomes, the ideal pacing modality in patients with ATTR-CM remains unclear, and uncertainty remains whether ATTR-CM disease stage should also be considered. It has previously been shown that CRT implantation, particularly in patients fulfilling current CRT implantation criteria, may improve NYHA functional class, LVEF, and survival in ATTR-CM.^12^^,^^13^ Small studies have suggested the feasibility and safety of LBBAP for patients with ATTR-CM.^14^^,^^15^ In the largest study to date (n = 23), one-third of patients fulfilled criteria for CRT implantation; however, neither in the overall cohort nor in patients with an LVEF of <50% (69% of the study population), a significant improvement in LVEF or NT-proBNP levels was observed, despite a significant reduction in paced QRS duration (LBBAP 122.8 ± 25.7 ms) compared with intrinsic QRS width (144.9 ± 38.8 ms).^15^ Despite conflicting evidence, recent case reports suggest a potential for functional and cardiac biomarker improvement with LBBAP in patients with ATTR-CM with symptomatic atrial fibrillation, even in patients with narrow QRS width.^16^

When comparing mortality and a combined end point of HF hospitalizations and device upgrade over all 3 pacing modalities, no significant differences were observed in our cohort (*P* = .38 [all-cause mortality]; *P* = .23 [HF hospitalization or device upgrade]) (Figure 4). Yet, in line with a higher proportion of patients diagnosed with ATTR-CM and HF before CRT and CSP implantation, annualized HF hospitalizations were more common in the CRT and CSP cohorts (24.1% [VVI/DDD]; 69.7% [CRT]; 76.7% [CSP]). In our cohort, neither NYHA functional class nor NT-proBNP levels significantly worsened after VVI/DDD PM implantation; however, a significant decline in renal function was seen (*P* = .02) (Table 7). As previously described with high RV pacing burden,^3^ we also observed a nonsignificant trend toward LV and RV functional decline and a significant increase in moderate-to-severe mitral valve regurgitation (n = 6 [19%] vs 27 [90%]; *P* < .001). CRT (n = 6) or LBBAP upgrade (n = 1) was performed in 7 patients, of whom 4 had received the PM prior to their ATTR-CM diagnosis, after presenting with atrial fibrillation (n = 6 of 7) and HF (n = 6 of 7) during follow-up. In CRT candidates, an improvement in NYHA class in line with previous findings^3^ was observed, together with a numerical improvement in NT-proBNP and LV and RV function. Unlike previous studies, an increase in moderate/severe mitral regurgitation was seen after CRT implantation. Despite achieving the shortest QRS width, a measure of electrical resynchronization, CSP resulted in a numerical increase in NT-proBNP and a small, but significant, reduction in renal function (*P* = .04).

After receiving a VVI/DDD PM for SSS or AV block, a ventricular pacing burden of >90% was seen in 40% of our patients immediately after device implantation and 65% at 3-month follow-up, respectively. A similar increase in ventricular pacing burden of >90% between implantation (n = 10 [45%]) and 3-month follow-up (n = 14 [64%]) was detected in patients presenting with PR prolongation at the time of implantation, before remaining stable thereafter. With many unknowns and evidence suggesting pacing burden to be an important determinant of clinical outcomes^3^—particularly in patients without a classical CRT indication—emphasis on optimizing PM settings irrespective of the pacing strategy may provide an opportunity to minimize unnecessary pacing, particularly in patients with PR prolongation or narrow QRS width.

Taken together, our study adds to the limited reported experience of cardiac pacing, resynchronization, and CSP in ATTR-CM and compares clinical outcomes after VVI/DDD, CRT, and LBBAP PM implantation for the first time. Despite the high clinical need, the optimal PM selection for cardiac pacing in this patient population remains unclear. It is currently not understood which patients may primarily benefit from cardiac resynchronization or LBBAP; whether improvement in functional class, LVEF, cardiorenal biomarkers, or clinical outcomes is achievable; and how intrinsic QRS morphology, presence of conduction abnormalities, and QRS duration should guide the decision to pursue cardiac pacing. Whether the benefit is reserved for patients with a current recommendation for CRT implantation or pacing indications may be expanded to patients with preserved LVEF, bradyarrhythmias, or insufficiently controlled atrial fibrillation (eg, pace and ablate strategy) needs to be explored. The knowledge gap around cardiac pacing provides an opportunity for future randomized trials to better inform and further improve the treatment of patients with ATTR-CM. Future studies need to investigate how to exploit the additional therapeutic opportunities provided by the implantation of physiological PM systems through modulating and optimizing pacing rate. The myPACE randomized clinical trial has provided initial, intriguing evidence suggesting the benefit of accelerated, individualized pacing in patients with HF and preserved ejection fraction, thereby challenging the long-held paradigm that patients with HF benefit from strict heart rate control at <70 beats/min.^17^ In individual cases, this approach has already been successfully adopted in patients with ATTR-CM.^18^

Finally, with the availability of disease-modifying therapies, emphasis has been put on the timely diagnosis of ATTR-CM to limit complications seen with advanced disease. Bearing this in mind, PM implantation in patients with increased LV wall thickness may offer an opportunity for earlier diagnosis, before the development of HF,^9^ as low RV and LV electrode sensing at implantation should raise suspicion and potentially referral to test for underlying ATTR-CM.

### Limitations

Numerous biases complicate the interpretation of our findings, owing to the retrospective, nonrandomized, unblinded study design. Treatment of ATTR-CM is rapidly changing, and TTR stabilizers, optimization of HF therapy, and improved rhythm management all contribute to the improvement in clinical outcomes over recent years. Follow-up data for the current study was collected between 2005 and 2024, a timespan during which diagnosis shifted from being typically biopsy based before 2017 to noninvasive in more than 80% of cases thereafter. Of patients implanted with a PM before the wider availability of TTR stabilizers, only 4% received tafamidis, with LVEFs for 2 CRT patients and 1 ICD patient on tafamidis remaining unchanged at 43% and 43.7%. Prescription rates for tafamidis were significantly higher in patients presenting from 2019 onwards, with 43%, 67%, and 79% in VVI/DDD, CSP, and CRT patients, respectively. Indications for cardiac pacing and selection of the pacing strategy were made at the discretion of the treating HF and heart rhythm specialist, taking the current available evidence into consideration. The higher risk for HF hospitalizations in patients implanted with CRT or CSP than VVI/DDD PMs in our cohort may in part reflect advanced ATTR-CM disease stages, while suggesting that there may be a role for conventional VVI/DDD pacing in patients with ATTR-CM without previous HF symptoms, particularly if stabilizing TTR-targeting therapy is timely initiated. Ascertainment bias is likely significant given that assessment of clinical status, laboratory, and echocardiographic parameters is not routinely performed during PM follow-up and thus often incomplete. Follow-up for these parameters is shorter than for clinical outcomes, and significant uncertainty remains regarding the long-term effect of pacing strategies. Complete follow-up is more likely in patients doing poorly and requiring care at referral centers. Although the study population is one of the largest single-center ATTR-CM cohorts requiring cardiac pacing, it was still small and overwhelmingly male. Conclusions about the ideal pacing strategy should not be made solely based on this study. The study was conducted at a single, tertiary referral center, limiting the external validity of the study findings.

## Conclusion

In patients with ATTR-CM requiring cardiac pacing, CSP was safe and led to shorter QRS width, indicative of better electrical cardiac resynchronization than conventional or CRT pacing, with stable lead performance over a 2-year follow-up period. The effect of pacing modalities on mechanical synchrony, cardiac remodeling, and clinical outcomes in ATTR-CM remains unclear and requires further study in randomized controlled trials.

## Disclosures

None of the authors has received any compensation for this study. Dr Dobner reports research grants from Österreichischer Herzfonds and speaker fees and travel grants from Alnylam, AstraZeneca, Bayer, Boehringer Ingelheim, and Pfizer outside of the submitted work. Dr Haeberlin has received travel fees/educational grants from Medtronic, Biotronik, Abbott, and Philips/Spectranetics without impact on his personal remuneration. He serves as a proctor for Medtronic. He has received research grants from the Swiss National Science Foundation, the Swiss Innovation Agency Innosuisse, the Swiss Heart Foundation, the University of Bern, the University Hospital Bern, the Velux Foundation, the Hasler Foundation, the Swiss Heart Rhythm Foundation, and the Novartis Research Foundation. He is a cofounder and chief executive officer of Act-Inno AG. Dr Gräni received funding from the Swiss National Science Foundation, InnoSuisse, Center for Artificial Intelligence in Medicine University Bern, Gambit Foundation, Novartis Foundation for Medical-Biological Research, Swiss Heart Foundation, Schmieder-Bohrisch Foundation, and Gottfried and Julia Bangerter-Rhyner Foundation, outside of the submitted work. Dr Gräni serves as editor-in-chief of The International Journal of Cardiovascular Imaging, Springer. Dr Gräni serves as a Congress Program Committee member of European Society of Cardiology 2024–2026. Dr Nitsche reports speaker/consulting honoraria from Pfizer, Bayer, Prothena, and Boehringer Ingelheim and research contracts with Pfizer, AstraZeneca, the Austrian Society of Cardiology, and the European Association of Cardiovascular Imaging. Dr Reichlin reports research grants from the Swiss National Science Foundation, the Swiss Heart Foundation, the sitem-insel support funds, Biotronik, Boston Scientific, and Medtronic, all for work outside the submitted study; speaker/consulting honoraria or travel support from Abbott/St. Jude Medical, Bayer, Biosense Webster, Biotronik, Boston Scientific, Farapulse, Medtronic, and Pfizer–Bristol Myers Squibb, all for work outside the submitted study; and support for his institution’s fellowship program from Abbott/St. Jude Medical, Biosense Webster, Biotronik, Boston Scientific, and Medtronic. All other authors report no conflicts.
