# Supplementary material for: Indication and electrical performance of conventional, resynchronization, and conduction system pacing in transthyretin amyloid cardiomyopathy
Source: Heart Rhythm O2. 2026 Mar 27;7(7):1289–301. doi: 10.1016/j.hroo.2026.03.026 (PMC13389980; doi:10.1016/j.hroo.2026.03.026)
Supplement: Supplementary Table 1 [file mmc1.docx]

**Supplemental Material**

Dobner et al.

Indication and electrical performance of conventional, resynchronisation and conduction system pacing in Transthyretin amyloid cardiomyopathy

ClinicalTrials.gov Identifier: NCT04776824

**Supplemental Table 1: Comparison of electrical performance between pacemaker systems at last follow-up**

| **Supplemental Table 1** Comparison of electrical performance between pacemaker systems at last follow-up | | | | |
| --- | --- | --- | --- | --- |
|  | **VVI/DDD group** n=35 | **CRT group**  n=14 | **CSP group**  n = 18 | **P-value** |
| **Available CIED follow-up duration [days]** | 1341 (881-2186) | 1084 (911-1501) | 714 (592-981) | **0.018** |
| **Electrode parameters during FU** |  |  |  |  |
| RA sensing [mV] | 1.7 (1.0-3.3) | 1.7 (0.75-7.5) | 2.3 (1.1-3.7) | 0.88 |
| RA threshold [V/0.4ms] | 0.75 (0.7-1.0) | 0.75 (0.7-0.8) | 0.90 (0.8-1.5) | 0.25 |
| RA impedance [Ω] | 460 (420-526) | 649 (480-684) | 527 (482-646) | 0.09 |
| RV sensing [mV] | 6.5 (4.7-8.7) | 5.7 (2.5-16.5) | 10.3 (7.8-16.5) | 0.20 |
| RV impedance [Ω] | 460 (410-519) | 531 (410-638) | 526 (468-550) | 0.09 |
| RV threshold [V/0.4ms] | 0.80 (0.6-1.0) | 0.70 (0.7-0.8) | 1.1 (0.75-1.4) | 0.07 |
| LV sensing [mV] | - | 10.7 (6.6-14.7) | 10.4 (9.0-13.8) | 1.0 |
| LV threshold [V/0.4ms] | - | 1.0 (0.76-1.25) | 1.0 (0.6-1.3) | 0.92 |
| LV impedance [Ω] | - | 674 (569-855) | 437 (344-546) | **0.003** |
| **Ventricular pacing during FU [%]** | 94 (69-99) | 99 (99-99) | 99 (95-100) | **0.028** |
| Values are presented as median (IQR). Comparisons between groups were made using the Kruskal-Wallis test or the Mann-Whitney U test as appropriate.  **Abbreviations:** LVEDD = left ventricular enddiastolic diameter; LVEF = left ventricular ejection fraction; MR = mitral valve regurgitation; NYHA = New York Heart Association; TR = tricuspid valve regurgitation.LV = left ventricle; RA = right atrium; RV right ventricle. | | | | |
